# Long-term follow-up in common variable immunodeficiency: the pediatric-onset and adult-onset landscape

**DOI:** 10.3389/fped.2023.1125994

**Published:** 2023-04-21

**Authors:** Maria Carrabba, Marco Salvi, Lucia Augusta Baselli, Serena Serafino, Marina Zarantonello, Elena Trombetta, Maria Cristina Pietrogrande, Giovanna Fabio, Rosa Maria Dellepiane

**Affiliations:** ^1^ Internal Medicine Department, RITA-ERN Center, Fondazione IRCCS Ca’ Granda Ospedale Maggiore Policlinico di Milano, Milan, Italy; ^2^ Department of Clinical Sciences and Community Health, Università degli Studi di Milano, Milan, Italy; ^3^ Pediatric Area, RITA-ERN Center, Fondazione IRCCS Ca’ Granda Ospedale Maggiore Policlinico di Milano, Milan, Italy; ^4^ Flow Cytometry Laboratory, Clinical Pathology, Fondazione IRCCS Ca’ Granda Ospedale Maggiore Policlinico, Milan, Italy

**Keywords:** common variable immunodeficiency (CVID), immune dysregulation, autoimmunity, lymphoid proliferation, cytopenia, CD21low B cells, inborn errors of immunity

## Abstract

**Introduction:**

The primary aim of this study is to investigate the evolution of the clinical and laboratory characteristics during the time in a longitudinal cohort of pediatric-onset and adult-onset Common Variable Immunodeficiency (CVID) patients in order to identify early predictive features of the disease and immune dysregulation complications.

**Methods:**

This is a retrospective-prospective monocentric longitudinal study spanning from 1984 to the end of 2021. The data of pediatric-onset vs. adult-onset patients have been compared for immunological features and for infectious and non-infectious complications assessed at diagnosis and follow-up.

**Results:**

Seventy-three CVID patients have been enrolled, with a mean of 10.0 years (SD ± 8.17) of prospective follow-up. At diagnosis, infections were observed in 89.0% of patients and immune dysregulation in 42.5% of patients. At diagnosis, 38.6% of pediatric-onset and 20.7% of adult-onset patients presented with only infections. Polyclonal lymphoid proliferation (62.1%) and autoimmunity (51.7%) were more prevalent in the adult-onset than in the pediatric-onset group (polyclonal lymphoid proliferation 52.3% and autoimmunity 31.8%, respectively). Enteropathy was present in 9.1% of pediatric-onset and 17.2% of adult-onset patients. The prevalence of polyclonal lymphoid proliferation increased during follow-up more in pediatric-onset patients (diagnosis 52.3%—follow-up 72.7%) than in adult-onset patients (diagnosis 62.1%—follow-up 72.7%). The cumulative risk to develop immune dysregulation increases according to the time of disease and the time of diagnostic delay. At the same age, pediatric-onset patients have roughly double the risk of having a complication due to immune dysregulation than adult-onset patients, and it increases with diagnostic delay. The analysis of lymphocyte subsets in the pediatric-onset group showed that CD21 low B cells at diagnosis may be a reliable prognostic marker for the development of immune dysregulation during follow-up, as the ROC curve analysis showed (AUC = 0.796). In the adult-onset group, the percentage of transitional B cells measured at diagnosis showed a significant accuracy (ROC AUC = 0.625) in identifying patients at risk of developing immune dysregulation.

**Discussion:**

The longitudinal evaluation of lymphocyte subsets combined with clinical phenotype can improve the prediction of lymphoid proliferation and allow experts to achieve early detection and better management of such complex disorder.

## 1. Introduction

Among the inborn errors of immunity, Common Variable Immunodeficiency (CVID) is considered the most common symptomatic primary antibody deficiency with an estimated prevalence between 1:25,000 and 1:50,000 inhabitants (1). CVID is caused by a primary defect in the development and/or functioning of the B cells, resulting in reduced serum levels of immunoglobulins (Ig) with reduced or absent specific antibody production (2–5). CVID clinical spectrum may be wide, and it is characterized predominantly by increased susceptibility to infections and/or by dysregulation of the immune system (1, 5). This background causes an extensive set of different clinical and immunological features with a broad range of comorbidities (6). The non-infectious complications may be evident at presentation or may appear afterward. They include autoimmunity, gastrointestinal inflammatory disease, liver disease, granulomatous lung disease, lymphoid hyperplasia and infiltrative disease, and the development of cancer, especially lymphoma (7, 8). Patients with CVID can be “categorized” into different phenotypes, according to their manifestations (7). This phenotypic distinction has important implications because the risk of death is estimated at 11 times higher for patients with non-infectious complications compared to those without (7, 8). The introduction of Ig replacement therapy has reduced the number of infections and improved their outcomes (9), but it does not seem to prevent or improve manifestations associated with immune dysregulation. CVID seems to have two different peaks of onset in the second or fourth decades of life, mainly between 20 and 45 years of age. The onset during childhood is usually recognized early by pediatricians if recurrent or severe infections are present. In adulthood, a diagnostic delay of 6–7 years (or later) after the onset of symptoms is often seen (10), perhaps due to the very heterogeneous nature of the disease and the importance of excluding secondary causes of hypogammaglobulinemia or lymphoproliferative disorders. This fact also causes a delay in accessing adequate therapy (11, 12), and, if associated with the higher prevalence of non-infectious complications, it contributes to worsening the prognosis of patients. Recently, non-infectious complications are emerging as the major challenge, not only in adult patients but also in the children, requiring a better understanding of pathogenesis and therapies (8). With this perspective, a number of monogenic defects have been recently identified in ∼10%–30% of CVID patients providing potential insights into both pathogenesis and additional therapeutics (8, 13).

The evolution of CVID disorder over time in patients diagnosed near to the onset of the disease, especially if diagnosed in childhood, has been rarely reported (14–16). A large cohort of patients and registry data have provided many insights into non-infectious complications of CVID, but the issue of early identification of patients with a high risk of developing complications or poor prognosis is still an open question (1, 7, 8, 10, 12, 14–17). The aim of this study is to investigate the evolution of the clinical and laboratory characteristics over time in a longitudinal cohort of pediatric-onset and adult-onset patients in order to identify early predictive features of disease complications. This study analyzes the organ-specific pathologies and the immunologic parameters identified in a monocentric cohort of patients with CVID since 1984 in a specialized referral center for the care of pediatric and adult patients with primary immunodeficiencies.

## 2. Materials and methods

### 2.1. Patients' selection

In this retrospective-prospective monocentric longitudinal study we have enrolled all patients with a diagnosis of Common Variable Immunodeficiency (CVID) who have been followed up since 1984 until December 2021 at the Primary Immunodeficiency referral Centre in Fondazione IRCCS Ca' Granda Ospedale Maggiore Policlinico, a tertiary and university care hospital in Milan, Italy. All the patients are enrolled in the European Society for Immunodeficiencies (ESID) Registry. The local Ethics Committee has approved this registry (Milan Area 2, Italy; ID # 509-2017). Each patient signed informed consent for data collection and scientific publications. Diagnosis of CVID was made based on current ESID diagnostic criteria (4, 18) or on the criteria of the original ESID/Pan American Immunodeficiency Group (PAGID) of 1999 (19). Diagnosis has also been reviewed and confirmed using the International Consensus Document (ICON) criteria (2). Among those patients originally diagnosed based on the ESID/PAGID criteria, this study included only the ones who had low levels of immunoglobulin G (IgG) and immunoglobulin A (IgA) and/or immunoglobulin M (IgM) plus poor antibody response to vaccines, or absent isohemagglutinin, and/or reduced values of switched memory B cells with CD4+ T cells above 200/µl. Clinical and immunological data were obtained from patients' medical records. For each patient, we excluded all secondary causes of hypogammaglobulinemia (20) and the presence of a marked cellular T deficiency.

### 2.2. Clinical and laboratory data

At the time of the study, eleven of the enrolled patients were still cared for at the pediatric center, and ten had been transferred to the adult center after reaching the age of 18 according to the transition path program at our hospital.

Documented clinical data include the clinical history of infections, bronchiectasis (confirmed with computed tomography), autoimmune cytopenias such as autoimmune hemolytic anemia (AIHA) and idiopathic thrombocytopenic purpura (ITP), organ-specific autoimmunity (including vitiligo, psoriasis, thyroid disease, atrophic gastritis), granulomatous disease, enteropathy, and malignancies. CVID-associated granulomatous interstitial lung disease (GLILD) was diagnosed based on typical CT scan results in the absence of evidence of an infectious or alternative cause and in a few cases confirmed by biopsy. Splenomegaly was defined as an increase in spleen size greater than or equal to 12 cm for adults, or above the age reference values for the pediatric population on ultrasound or CT or magnetic resonance (MRI), including the previous splenectomy of an enlarged spleen. Lymphadenopathy was detected on palpation, ultrasound, and CT or MRI. Granulomatous disease was defined by the finding of at least one granuloma not explained by other causes and demonstrated on biopsy, excluding granulomas associated with Crohn's disease. Enteropathy includes all cases of non-inflammatory bowel disease biopsy-proven infection (excluding ulcerative colitis and Crohn's disease) and hyperlymphocytosis (lymphocytic infiltration of the mucosa, lamina propria, and/or submucosa), defined as lymphocytic colitis. Clinical phenotypes were defined according to Chapel's et al. classification (7). Data on human polyclonal immunoglobulin (Ig) replacement therapy (RT) were recorded.

All patients underwent blood tests both at diagnosis and at the periodic follow-up because this investigation “at follow up” has been used as the latest data available. Malignant tumors include hematological malignancies and all other forms of cancer. The tumor diagnosis and staging were performed according to national guidelines currently in force. Gastric cancer was diagnosed with gastrointestinal endoscopy associated with biopsies. Breast cancer was diagnosed with standard imaging tests followed by histological confirmation. Lymphomas were diagnosed with imaging, including CT, MRI, and PET, as well as the histological examination of tissue sections, lymph nodes, and/or bone marrow biopsies, according to the current WHO classification. Diagnosis of non-melanocytic skin cancer (NMSC) was clinically performed and histologically confirmed after tumor excision. Patients were all treated and followed up according to current clinical practice at the time of their management.

### 2.3. Genetic analysis

Genetic analysis of all 73 patients was performed through sequencing of genes associated with CVID. The analyses were carried out at the Institute of Molecular Medicine Angelo Nocivelli of the ASST Spedali Civili of Brescia. The genetic study of 71 patients was performed using Next-Generation Sequencing (NGS) techniques on the Ion Torrent platform. Patients were screened through a target panel of 52 genes related to CVID and hypogammaglobulinemia. The identified mutations were then confirmed with Sanger sequencing and validated through those already described in the literature. One more patient was diagnosed with NGS performed by another laboratory (Center for Autoinflammatory Diseases and Immunodeficiencies, IRCCS Istituto Giannina Gaslini, Genova), and the last patient was diagnosed using Whole Exome Sequencing (WES) performed by the Laboratory of Medical Genetics of Papa Giovanni XXIII Hospital in Bergamo.

### 2.4. Definitions

The diagnostic delay was calculated from the age of the onset of CVID to the date of diagnosis. Follow-up time was considered to be the time from the date of CVID diagnosis to the last visit in December 2021. The disease time was defined as the time that elapsed between the age of the onset of the disease and the date of the last visit performed.

### 2.5. Statistical analysis

All the variables of interest were analyzed with descriptive statistics. Variables with continuous data were presented as mean and standard deviation (SD) and median values and their intervals. Categorical data were presented as frequencies and percentage values. Comparison of the means of continuous variables was performed with the appropriate tests for paired or independent samples, according to Levene's test for variance. Categorical variables were compared with Fisher's exact test. Sensitivity and specificity analysis of the predictive value of continuous variables of interest was performed using Receiver Operating Characteristic (ROC) curves. The correlation coefficient of Spearman was used to analyze the relationships between continuous and/or dichotomous variables. All analyses were performed with IBM SPSS Statistics (SPSS Inc. Chicago, IL, version 27.0). Graphs were made with Windows Excel and SPSS, and the graphs of lymphocyte subpopulations were made with GraphPad Prism version 9.0 for Windows (GraphPad Software, La Jolla, California).

## 3. Results

**Figure 1:**
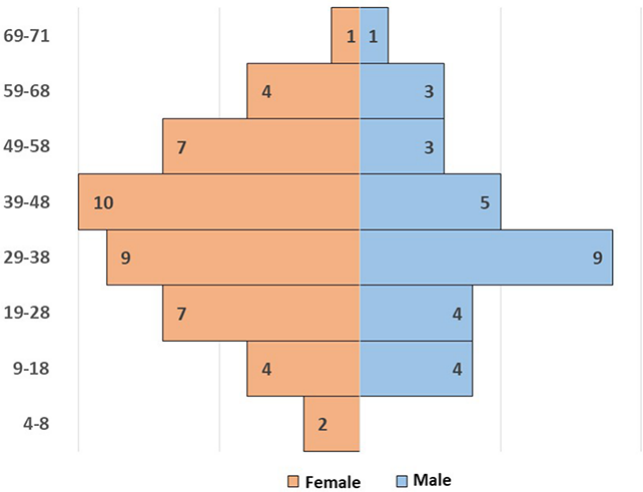
Demographic distribution of age and sex of the whole CVID cohort.

A total of 78 patients were initially diagnosed with CVID from 1984 to 2021. The diagnosis was revised and five patients were excluded: one was diagnosed with X-linked Agammaglobulinemia (XLA) after NGS analysis (pathogenic mutation on BTK gene); one could not be followed up; three did not fulfill the diagnostic criteria for CVID. Finally, this study enrolled 73 patients, 29 men and 44 women (1:1.5) (Figure 1).

All patients were of Italian origin except four patients who were from Egypt, Colombia, Albania, and Ukraine. There was a case of consanguinity of parents, first cousins, in one pediatric patient; two patients were familial cases of CVID. In 2017 two patients were lost; one died of post-traumatic cerebral hemorrhage at the age of 71, and 34-year-old patient was transferred to another center after 17 years of follow-up.

At the time of this study, patients were aged between 4 and 71 years (mean age 38.4 years ± 16.59). The mean age of CVID onset was 17.3 years ± 13.84 (range <1 to 63 years), but the mean age at diagnosis was 28.3 years ± 15.94 (range 3 to 67 years) with a mean diagnostic delay of 11.5 years ± 11.71 (median 6.0 years and range <1 to 47 years). The mean of the estimated time of disease was 21.5 ± 13.86 years (range 1 to 59 years) with a mean time of prospective follow-up of 10.0 years ± 8.17 (See Table 1). All the patients, except one who refused, were under replacement therapy with intravenous (52.8%) or subcutaneous (47.2%) immunoglobulins.

**Table 1:** Demographic characteristics, features at CVID onset and diagnosis are detailed.

	Overall CVID cohort (*N* = 73)		Pediatric-onset (*N* = 44)		Adult-onset (*N* = 29)		
Demographic Characteristics	Mean	SD	Mean	SD	Mean	SD	*p*
Age at the latest follow-up	38.4	16.59	42.9	13.45	47.4	12.89	0.213
Age at onset	17.3	13.84	10.3	6.68	30.8	11.29	<0.001
Age at diagnosis	28.4	16.00	31.6	12.17	38.9	12.59	0.041
Diagnostic delay	11.5	11.71	21.7	13.76	8.2	7.76	<0.001
Follow up time	10.0	8.17	11.2	9.45	8.6	7.67	0.279
Time of disease	21.5	13.86	33.0	15.38	16.8	10.23	<0.001
Characteristics at CVID onset	*N*	%	*N*	%	*N*	%	*p*
Immune Dysregulation	31.0	42.47	16.0	36.36	15.0	51.72	0.231
Infections	65.0	89.04	38.0	86.36	27.0	93.10	0.465
Malignancy	0.0	0.00	0.0	0.00	0.0	0.00	NA
Syndromic Features	3.0	4.11	3.0	6.82	0.0	0.00	0.272
Other Symptoms	5.0	6.85	3.0	6.82	2.0	6.90	1
No Symptoms	2.0	2.74	2.0	4.55	0.0	0.00	0.514
Hypogammaglobulinemia	2.0	2.74	2.0	4.55	0.0	0.00	0.514
Characteristics at CVID diagnosis	*N*	%	*N*	%	*N*	%	*p*
Recurrent respiratory infections	65	89.0	42	95.5	24	82.8	0.252
Failure to thrive (infancy)	5	6.8	5	11.4	0	0.0	0.51
Recurrent pyogenic infections	6	8.2	2	4.5	4	13.8	0.207
Unusual infections	7	9.6	4	9.1	3	10.3	ns
Same pathogen recurrent infections	7	9.6	5	11.4	2	6.9	0.696
Autoimmunity, autoinflammation, and lymphoid proliferation	30	41.1	16	36.4	14	48.3	0.34
Pneumonia	44	60.3	26	59.1	18	62.1	ns
Meningitis	6	8.2	3	6.8	3	10.3	0.676
Upper respiratory airways recurrent infections	64	87.7	41	93.2	23	79.3	0.142
Lower respiratory airways recurrent infections	40	54.8	22	50.0	18	62.1	0.345
Gastrointestinal recurrent infections	25	34.2	16	36.4	9	31.0	0.802
Urinary tract infections	14	19.2	6	13.6	8	27.6	0.223
Skin recurrent infections	6	8.2	3	6.8	3	10.3	0.676
Other recurrent infections	4	5.5	2	4.5	2	6.9	ns
Unusual viral infections	11	15.1	7	15.9	4	13.8	ns
Unusual opportunistic infections	4	5.5	2	4.5	2	6.9	ns
Unusual parasitic infections	1	1.4	0	0.0	1	3.4	0.394
Bronchiectasis	31	43.1	18	41.9	13	44.8	0.813
Enteropathy	9	12.3	4	9.1	5	17.2	0.469
Lymphocytic colitis	9	12.3	4	9.1	5	17.2	0.469
Hepatomegaly	16	21.9	8	18.2	8	27.6	0.394
Splenomegaly	38	52.1	21	47.7	17	58.6	0.474
Liver disease	5	6.8	3	6.8	2	6.9	ns
Pulmonary disease	29	39.7	18	40.9	11	37.9	ns
GLILD	1	1.4	0	0.0	1	3.4	0.397
Other organ diseases	10	13.7	7	15.9	3	10.3	0.73
Hypothyroidism	3	4.1	2	4.5	1	3.4	ns
Chronic diarrhea	5	6.8	4	9.1	1	3.4	0.642
Autoimmune cytopenia	17	23.3	10	22.7	7	24.1	ns
Autoimmune hemolytic anemia	8	11.0	6	13.6	2	6.9	0.465
Autoimmune neutropenia	3	4.1	1	2.3	2	6.9	0.559
Immune Thrombocytopenia	17	23.3	10	22.7	7	24.1	ns
Evans syndrome	9	12.3	6	13.6	3	10.3	ns
Other organ autoimmunity	13	17.8	6	13.6	7	24.1	0.35
Thyroiditis	7	9.6	3	6.8	4	13.8	0.425
Atrophic gastritis	1	1.4	1	2.3	0	0.0	ns
Vitiligo	2	2.7	2	4.5	0	0.0	0.514
Psoriasis	3	4.1	1	2.3	2	6.9	0.559
History of malignancy	1	1.4	0	0.0	1	3.4	0.397
Lymphoid Nodular Hyperplasia	7	9.6	4	9.1	3	10.3	ns
Granulomas	3	4.1	1	2.3	2	6.9	0.559
Celiac disease	5	6.8	2	4.5	3	10.3	0.38
Allergy	16	22.2	12	27.9	4	13.8	0.248
Persistent HCV viremia	0	0.0	0	0.0	0	0.0	na
Persistent HIV viremia	0	0.0	0	0.0	0	0.0	na
Persistent EBV viremia	3	4.8	1	2.6	2	8.0	0.557
Persistent CMV viremia	1	1.6	1	2.8	0	0.0	ns
Pathologic HRCT	51	79.7	32	82.1	19	76.0	0.751
Pathologic endoscopy	23	63.9	14	77.8	9	50.0	0.164

Table 1 shows that the most frequent clinical manifestation of CVID at onset was recurrent infection (89.0%), especially recurrent respiratory infections, followed by immune dysregulation (42.5%). Five patients had other symptoms (chronic diarrhea, eczema, liver failure, short stature, and asthenia), two patients were asymptomatic, and three patients had associated syndromic features. After the diagnosis, when NGS target panel was available, all the patients underwent genetic analysis. Nineteen patients (26.0%) were found to be carriers of a monogenic pathogenic defect: nine on TNFRSF13B (TACI), two on CTLA-4, one on LRBA, ADA2, TWE-PRIL, CXCR4, CD40, TTC37, NFKB1, and ATP6AP1 gene (Supplementary Table S1). The small number of patients does not allow for correlating the genetic result with the phenotype in immune dysregulation in particular. We found that among the 19 patients with the identified gene, the “only infections” phenotype was present in four (21%) and the other 15 had CVID complications (autoimmunity, splenomegaly, polyclonal lymphoid proliferation, and noninfectious enteropathy). Among the 54 patients with no gene identified, 13 (24.1%) had the “only infections” phenotype, while all the other 41 presented complications.

**Figure 2:**
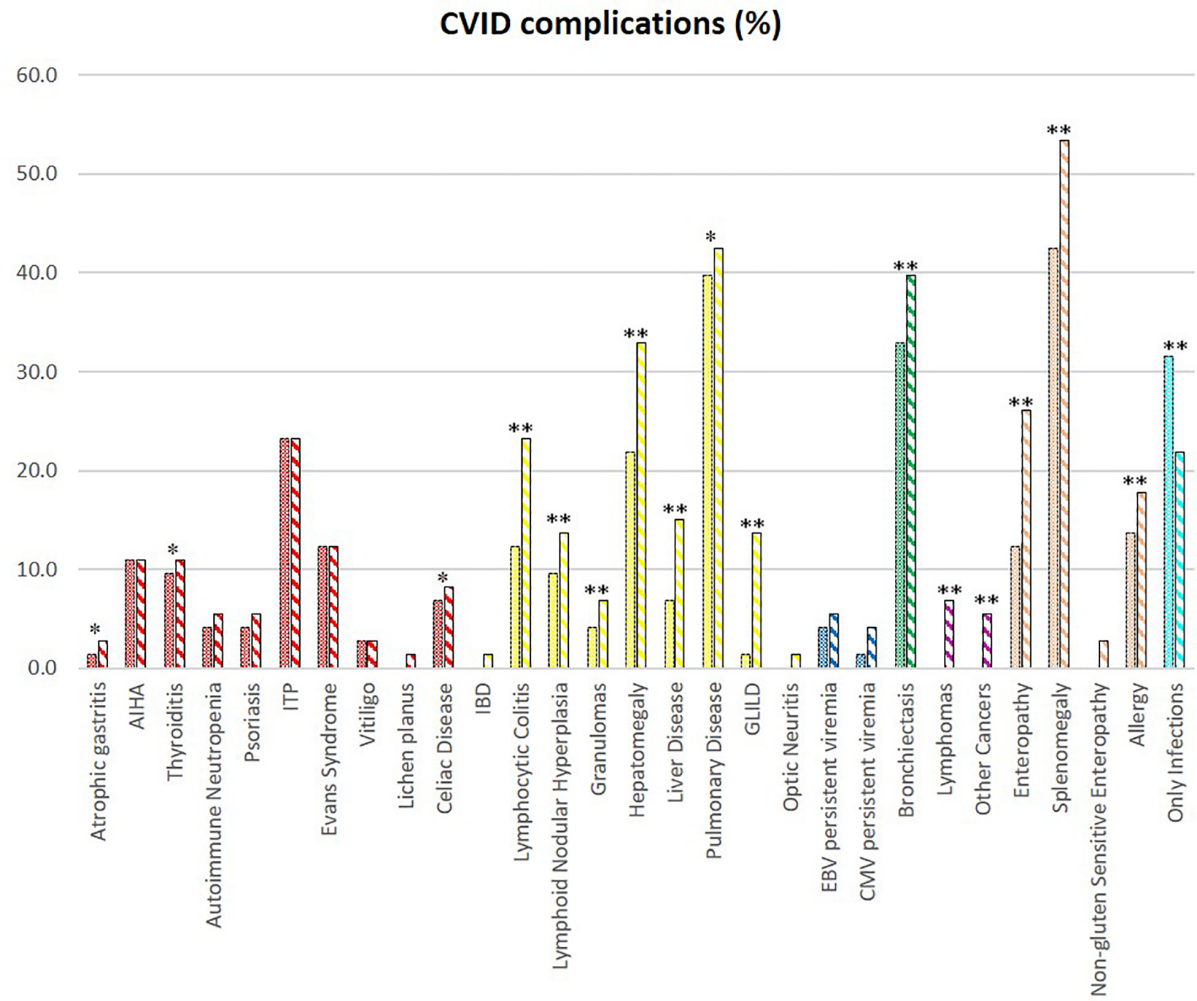
CVID complications of the whole cohort. The comparison between manifestations at diagnosis (full colored) and follow-up (colorful stripped) is shown if significate: **p < 0.001; *p < 0.05.

We evaluated the overall cohort's disease complications at diagnosis and follow-up (Figure 2). Over time, there is an increase in the prevalence of bronchiectasis (*p* < 0.001), splenomegaly (*p* < 0.001), hepatomegaly (*p* < 0.001), and enteropathy (*p* < 0.001). There was an overall increase in immune dysregulation features, such as lymphocytic colitis (*p* < 0.001), nodular lymphoid hyperplasia (*p* < 0.001), granulomas (*p* < 0.001), granulomatous lymphocytic interstitial lung disease (GLILD) (*p* < 0.001), and granulomatous liver disease (*p* < 0.001). Lymphomas arose during follow-up in five patients in adult age.

### 3.1. Analysis according to age at CVID onset

Patients were divided into a “pediatric-onset cohort”, as defined by age of onset of CVID from 2 to 18 (*n* = 44), and an “adult-onset cohort”, as defined by age of onset of disease >18 years old (*n* = 29). Early onset (age <11) is prevalent in men, while a later onset was observed in women.

**Figure 3:**
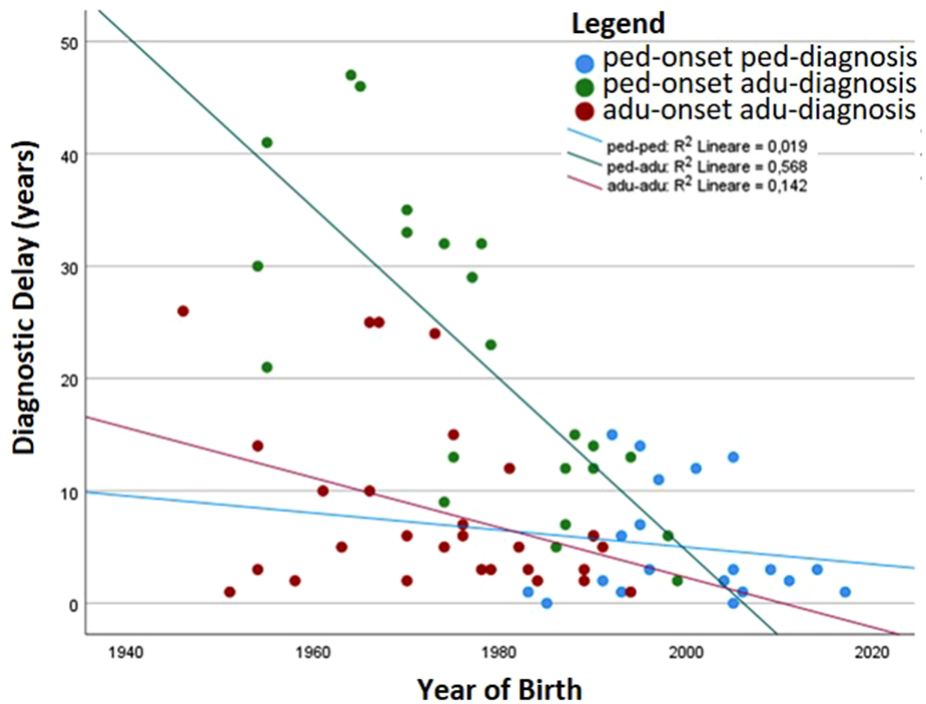
The patients’ year of birth has been plotted with the time of diagnostic delay. The correlation index (*R*^2^) is also shown.

Table 1 details the age at diagnosis and follow up and time of disease and follow up between the two groups. Comparison between the two groups shows that the diagnostic delay is higher in the pediatric-onset rather than in the adult-onset group (*p* = 0.031), and both groups have been followed up at our centers for a similar follow-up time. We analyzed the distribution of patients by year of birth and the time of diagnostic delay considering that before the 1990s CVID disorders were poorly known in Italy. Indeed, the difference between the year of birth and diagnostic delay is less evident for pediatric-onset patients with a diagnosis in the pediatric age (R2 linear = 0.019) and for adult-onset patients with a diagnosis in adulthood (R2 linear = 0.142) (Figure 3). For patients with pediatric-onset and diagnosis in adulthood, we can observe the inversely proportional correlation between the two parameters (R2 linear = 0.568) since several patients were born before 1980. Analyzing other differences in diagnostic delay, the subgroup of patients with pediatric-onset and pediatric diagnosis (*n* = 21) had the CVID onset very early, before the age of 5. On the other hand, patients with pediatric-onset but diagnosed in adulthood (*n* = 23) had the CVID onset at the beginning of adolescence (age >11). Failure to thrive was one of the factors that probably led to an earlier diagnosis in the former group than in the latter (*p* = 0.019). At follow-up, patients with pediatric-onset and adult-diagnosis manifested gastrointestinal complications more frequently than the patients with pediatric diagnosis (gastrointestinal infections: *p* = 0.011; colitis lymphocytic: *p* = 0.023), and the endoscopy was found to be pathological in a higher rate of patients with pediatric-onset and adult-diagnosis than in the ones with pediatric-diagnosis (*p* = 0.038).

### 3.2. Clinical and laboratory characteristics at diagnosis and follow-up

Initial clinical manifestations observed at onset in the two groups of pediatric-onset and adult-onset patients are detailed in Table 1. Infections and immune dysregulation were observed to play the predominant role, but no statistically significant differences were found between the two groups. The analysis of infectious complications at diagnosis shows that recurrent upper and lower respiratory tract infections are predominant in the two groups, but there are no significant differences between them. The longitudinal analysis showed that the two groups have an equal incidence of infectious manifestations during follow-up, except for urinary tract infections which are more frequent in adult-onset patients (*p* = 0.021) (Supplementary Table S2). At diagnosis, bronchiectasis was already present in 40.9% of pediatric-onset patients and 44.8% of adult-onset patients with no differences between the two groups. Allergic manifestations were found in 12 pediatric-onset patients and 4 adult-onset patients with a similar prevalence.

**Figure 4:**
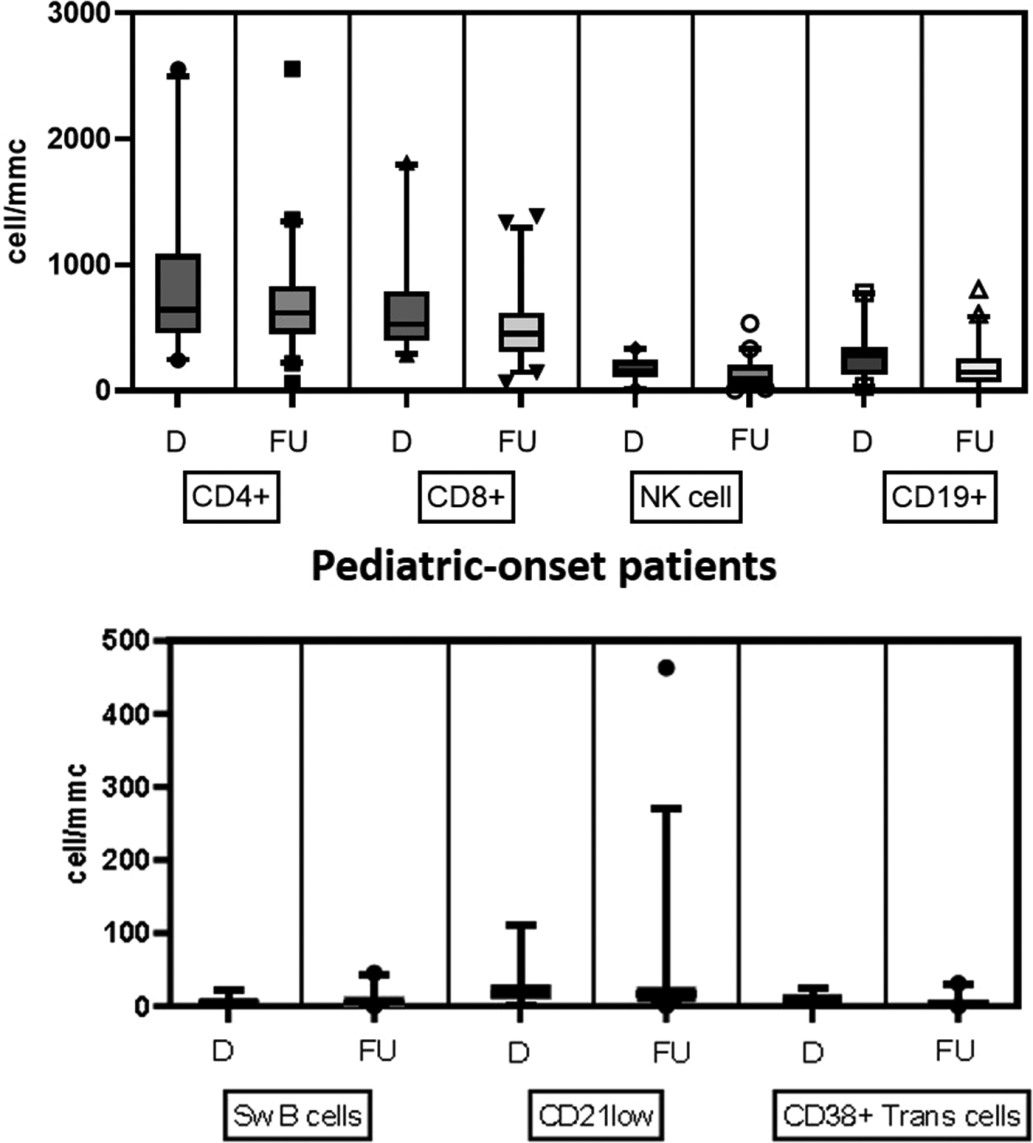
Distribution of B, T and NK cell subsets, switched memory B cells, CD21 low B cells, and transitional B cells at diagnosis and follow up in the pediatric-onset group.

**Figure 5:**
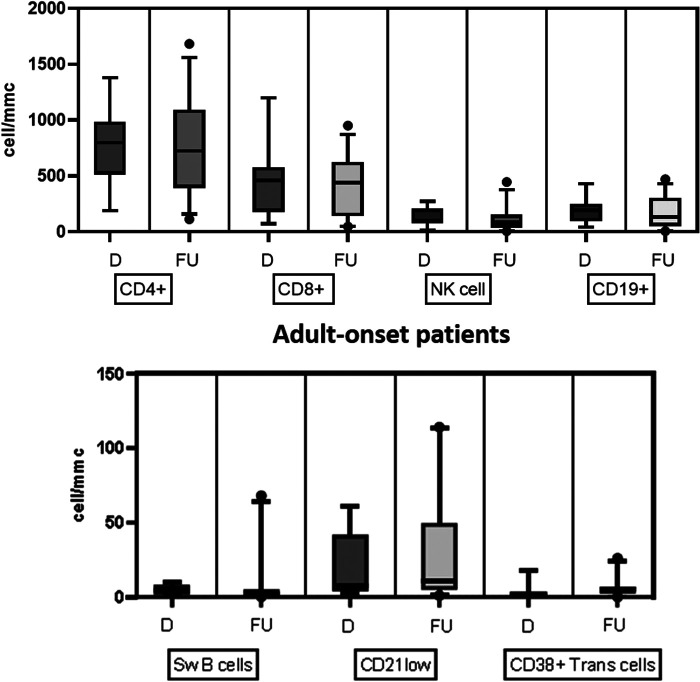
Distribution of B, T and NK cell subsets, switched memory B cells, CD21low B cells, and transitional B cells at diagnosis and follow up in the adult-onset group.

Table 2 details the serum level of IgA, IgG, and IgM and cell subsets CD19+, and switched memory B cells at diagnosis in the two groups. The lymphocyte subpopulations were further paired and compared between diagnosis and follow-up for each age group onset, evaluating CD4+ and CD8+ lymphocytes, NK cells, CD19+ lymphocytes, switched memory B cells, CD21 low B cells, and transitional B cells (Figures. 4, 5). In the pediatric-onset group, the transitional B cells significantly decreased at follow-up (*p* = 0.021), while the other cell subsets did not change over time, although the CD21 low B cells appeared to have increased at follow-up (Figure 4). In the adult-onset group, the paired analysis between diagnosis and follow-up revealed no differences, also in this group, CD21 low B cells tended to increase over time (Figure 5).

**Table 2:** Laboratory findings at CVID diagnosis.

	Overall CVID cohort (*N* = 73)		Pediatric-onset (*N* = 44)				Adult-onset (*N* = 29)	
			Pediatric-onset/ pediatric-diagnosis (*N* = 21)		Pediatric-onset/ adult-diagnosis (*N* = 23)			
Laboratory Characteristics	Mean	SD	Mean	SD	Mean	SD	Mean	SD
IgG (mg/dl)	203.6	148.68	240.3	139.34	313.0	148.43	174.8	151.85
IgM (mg/dl)	11.4	18.19	22.5	18.12	28.5	24.50	18.8	19.37
IgA (mg/dl)	20.5	18.74	6.3	6.11	54.8	173.03	15.4	23.11
CD19+ B cells (cell/µl)	215.2	111.46	238.6	103.30	373.3	284.16	210.7	119.24
Switched memory B cells (%)	3.1	2.07	2.5	1.72	5.8	6.31	3.5	2.17

### 3.3. Clinical phenotypes

**Figure 6:**
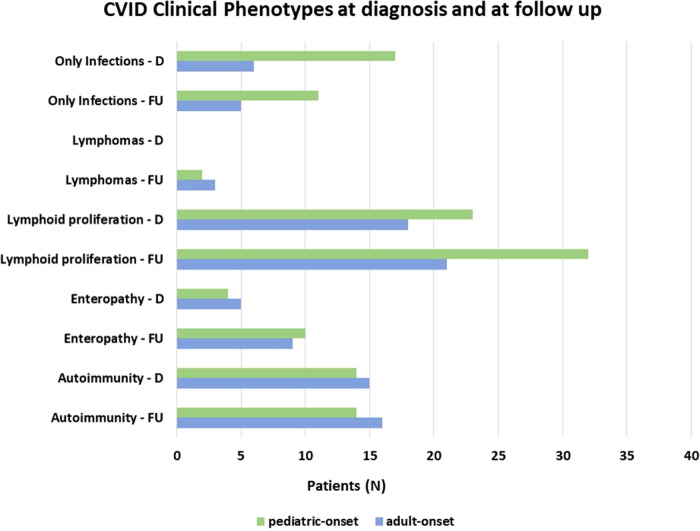
Distribution of patients according to Chapel et al clinical phenotypes (7) at diagnosis and follow-up in the pediatric-onset and adult-onset groups.

**Figure 7:**
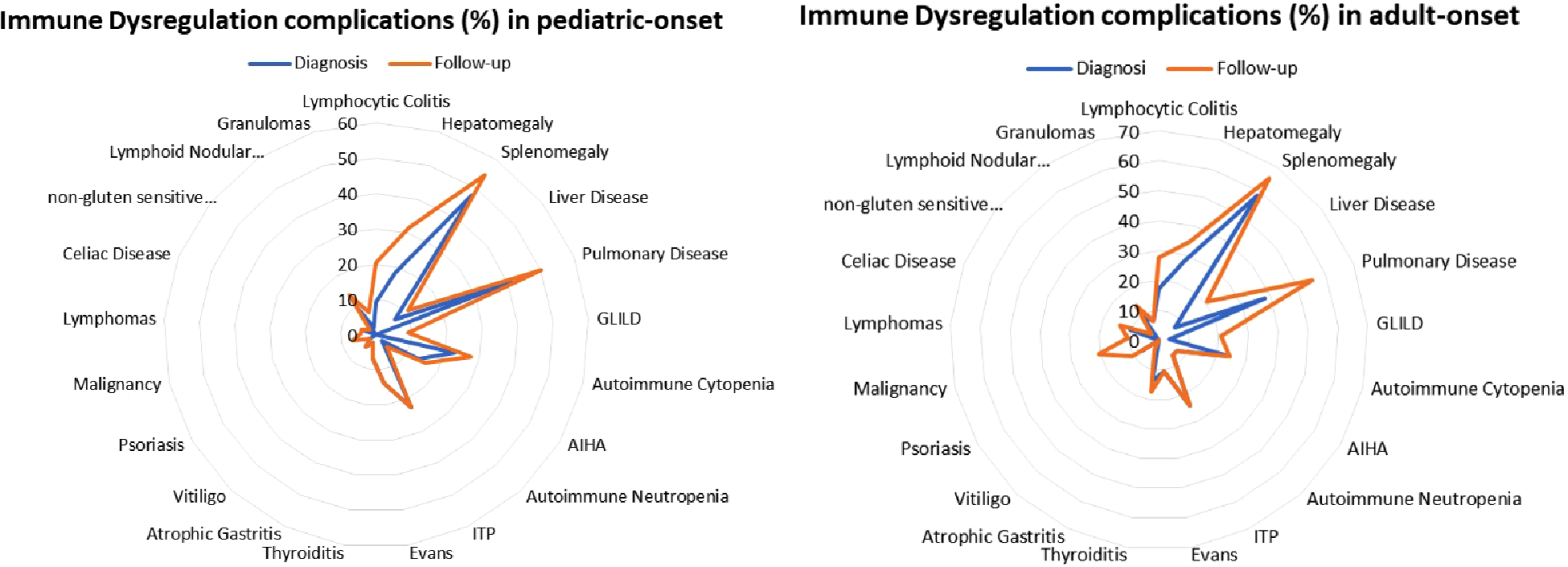
Prevalence of complications of immune dysregulation at diagnosis and at follow-up in the pediatric-onset (**A**) and adult-onset patients (**B**).

Patients were assigned one of the five clinical phenotypes according to the ones by Chapel et al. (7) at diagnosis and follow-up. Figure 6 shows findings, while Table 1 and Supplementary Table S2 detail data. According to the phenotypes' categorization at follow-up, the pediatric-onset group has the main changes in complications, reducing the number of patients who presented with only infections. Complications were due to immune dysregulation as polyclonal lymphoid proliferation and enteropathy increased. In the adult-onset group, the phenotype seemed not to change between onset and follow-up, but lymphoma with a rate of 10.3% at follow-up appeared as the main complication of immune dysregulation. Manifestations of immune dysregulation at diagnosis and follow-up are represented in Figure 7. Polyclonal lymphoid proliferation represents the group of complications most developed over time. The prevalence of splenomegaly is high at diagnosis in both groups (40.9% pediatric-onset and 44.8% adult-onset) and increased considerably even at the last follow-up (52.3% pediatric-onset and 55.2% adult-onset). Nodular lymphoid hyperplasia is found at diagnosis in 9.1% pediatric-onset and 10.3% adult-onset, and at follow-up in 13.6% pediatric-onset and 13.8% adult-onset. Granulomas are found at diagnosis in 2.3% pediatric-onset and 6.9% adult-onset and increased at follow up in the pediatric-onset group at 6.9%; one more pediatric-onset patient had granuloma with optic neuritis (21). Pulmonary lung disease, including granulomatous lymphocytic infiltration (GLILD) and thoracic lymphadenopathy, is the most common organ-specific complication both in patients with pediatric-onset (45.5%) and with adult-onset (37.9%). GLILD was already present at the diagnosis of CVID only in one adult-onset patient (1.4%), but its prevalence greatly increases at the last follow-up to 20.7% of adult-onset and 9.1% of pediatric-onset.

### 3.4. Analysis of the risk for immune dysregulation

**Figure 8:**
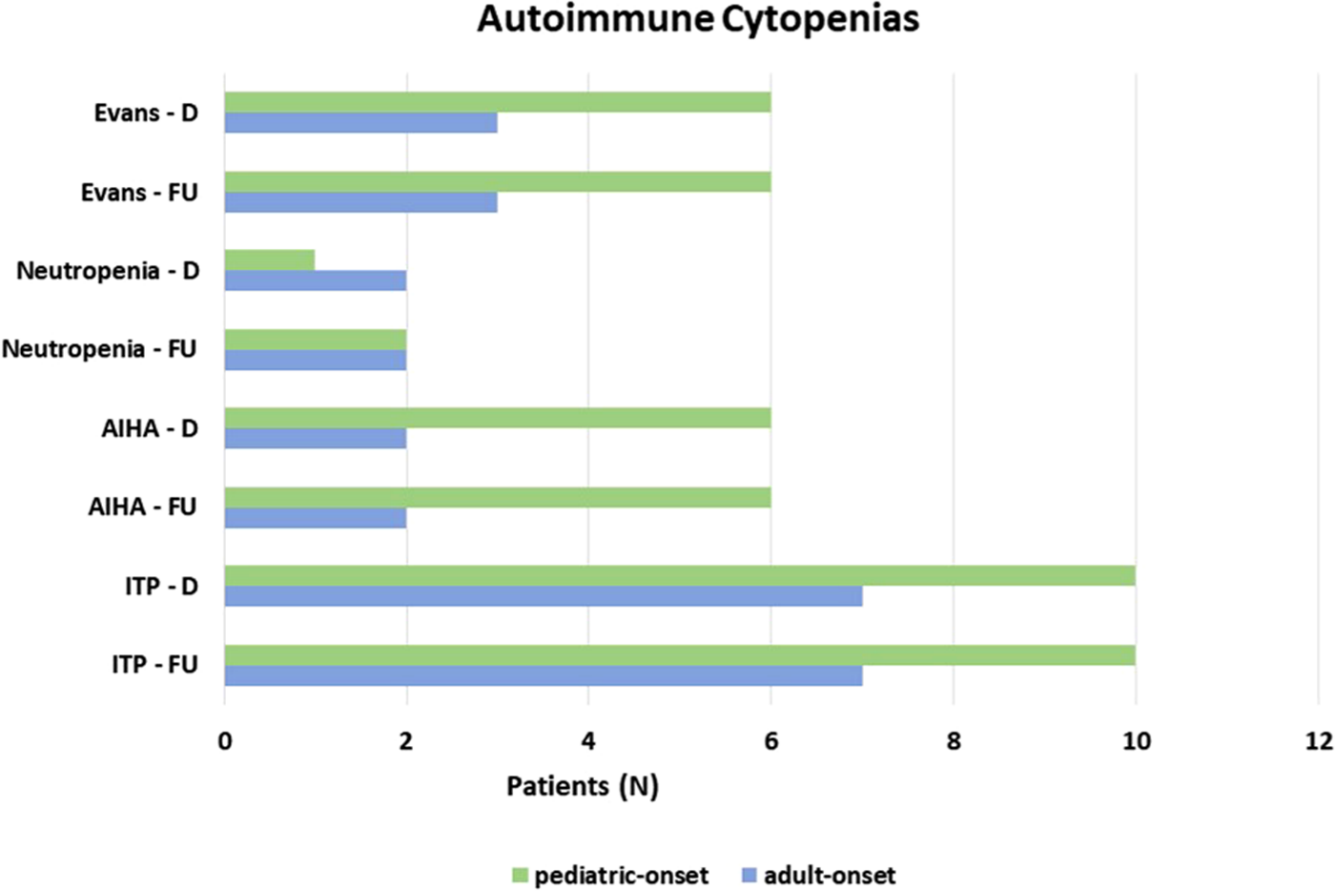
Prevalence of autoimmune cytopenias at diagnosis and at follow up in the pediatric-onset and adult-onset groups.

Autoimmune complications, including autoimmune cytopenias and/or organ-specific autoimmunity, were seen at diagnosis in 41.1% of the overall cohort. All the patients with autoimmune cytopenias (23.3%) presented immune thrombocytopenic purpura (ITP), 22.7% of pediatric-onset and 24.1% of adult-onset. Associated with autoimmune hemolytic anemia (AIHA) in 11% of overall subjects and with autoimmune neutropenia in 5.5% of overall patients in the context of Evans syndrome is 12.3% of the whole cohort. There was no change from diagnosis to follow-up on the prevalence of autoimmune cytopenias in both pediatric-onset and adult-onset groups (Figure 8). Table 1 and Supplementary Table S2 detail findings for organ-specific autoimmunity. Enteropathy at diagnosis was documented in 9.1% of pediatric-onset patients and 17.2% of adult-onset patients. Its prevalence increases considerably at the last follow-up both in patients with pediatric-onset (22.7%) and in patients with adult-onset (31.0%). Lymphocytic colitis affects 20.5% of pediatric-onset and 27.6% of adult-onset patients, furthermore, gluten non-sensitive enteropathy has been diagnosed in 2.3% of pediatric-onset and 3.4% of adult-onset patients. Hepatomegaly was found at diagnosis in 40.9% of pediatric-onset and 44.8% of adult-onset patients, and its prevalence increased during follow-up reaching 52.3% of pediatric-onset and 55.2% of adult-onset patients. Liver disease has been documented at the last follow-up in 21.7% of pediatric-onset and 37.5% of adult-onset patients who have hepatomegaly. As for malignancy, no patients had a history of cancer at diagnosis. Neoplasms occurred during follow-up in 12.3% of the whole cohort, including gastric adenocarcinoma (2 patients), non-melanocytic skin cancer (1 patient), breast cancer (1 patient), and lymphomas (5 patients, 2 in the pediatric-onset and 3 in the adult-onset group). Two were non-Hodgkin's lymphomas, and three were EBV-related lymphomas: one Burkitt's lymphoma and two Hodgkin's lymphomas. These both occurred in patients with pediatric-onset CVID which was complicated by relapsing and refractory autoimmune cytopenia. NGS identified a pathogenic mutation on CTLA-4 gene for both patients. The estimated risk of malignancies in patients affected with CTLA-4 deficiency is approximately 12.9%. The reported cases are mostly EBV-positive lymphomas (HL, DLBCL, and BL) (22). The patient with Burkitt's lymphoma had acute EBV infection documented 5 years before the lymphoma. Splenomegaly, lymphadenopathy, and chronic EBV viremia (very low copies circulating) were persistent for two years before lymphoma. NGS identified two mutations on the TACI gene associated with CVID (23).

### 3.5. Analysis of the risk for immune dysregulation

**Figure 9:**
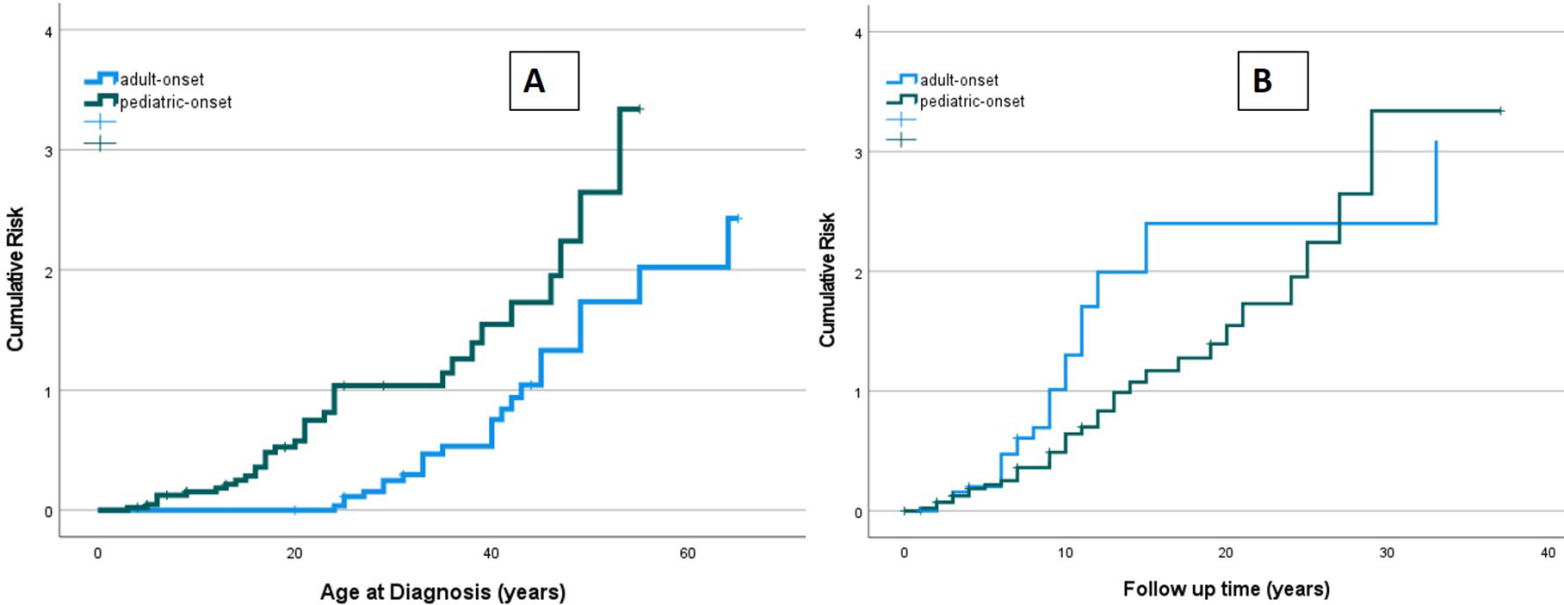
Cumulative risk curves of developing immune dysregulation observed in the pediatric-onset group (green line) and the adult-onset group (blue line) according to age at diagnosis of CVID (**A**) and time of CVID follow-up (**B**).

The risk of developing at least one complication during follow-up due to immune dysregulation was assessed considering the age at the onset of CVID (Figure 9A). At the same age, pediatric-onset patients have about twice the risk of having complications due to immune dysregulation than adult-onset patients. The risk is enhanced in pediatric-onset adult-diagnosis patients, and it increases with diagnostic delay. Considering the time of follow-up since the diagnosis, the early years' patients with pediatric-onset and adult-onset seem to have an overlapping risk of having immune dysregulation features (Figure 9B), as they may have those just present at diagnosis. After the first ten years of follow-up, adult-onset patients seem to have double the risk of developing a complication due to immune dysregulation compared to pediatric-onset patients.

**Figure 10:**
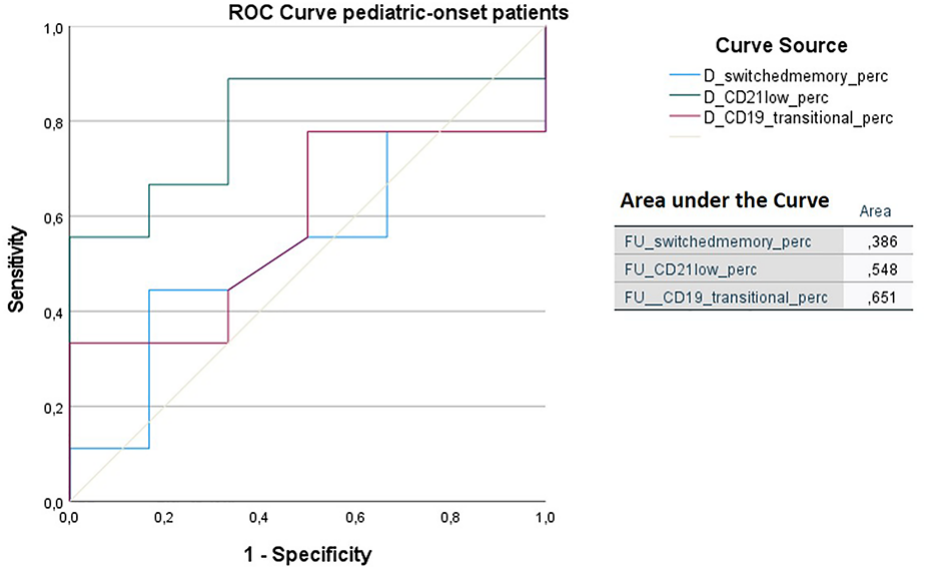
ROC curve analysis to predict immune dysregulation at follow-up of switched memory B cells (%), CD21low cells (%), and transitional B cells (%) measured at diagnosis in the pediatric-onset group. Details of the area under the curve (AUC) on the graph.

In order to identify biomarkers predictive of immune dysregulation development during follow-up, some indices were evaluated based on the data reported in the literature (24–26). In particular, the predictive power of the CD21low B cells assessed at diagnosis to identify subjects who would develop immune dysregulation at follow-up was analyzed by ROC curve. If we analyze the entire cohort of 73 patients with CVID, the area under the curve of CD21low cells is not significant. Considering the pediatric-onset group, the percentage of CD21 low B cells at diagnosis may be a reliable prognostic marker for the development of immune dysregulation during follow-up as the ROC curve analysis showed (AUC = 0.796), illustrated in Figure 10. The sample was numerically small (*n* = 14), therefore the identified cut-off value (>3%) is lower than the known data in the literature (>10%) (24). In the group of patients with adult-onset, the very small sample size (*n* = 8) of CD21 low B cell data at diagnosis greatly limits the analysis of the ROC curve (data not shown). Another index evaluated was the percentage of transitional B cells measured at diagnosis, which showed significant accuracy (ROC AUC = 0.625) in identifying adult-onset patients at risk of developing immune dysregulation, despite the limitation of the small sample (data not shown).

**Figure 11:**
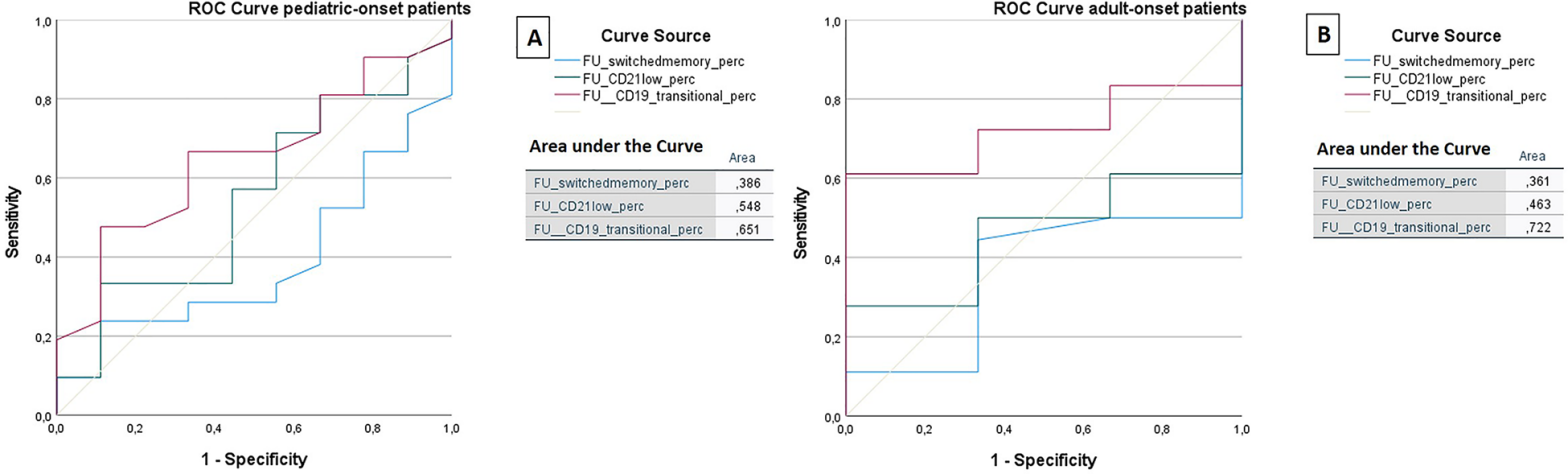
ROC curve analysis to predict the presence of immune dysregulation of switched memory B cells (%), CD21low cells (%), and the transitional B cells (%) measured at follow-up in the pediatric-onset group (**A**) and adult-onset (**B**) group.

The ROC analysis of the same cell subsets at follow-up shows that in both the pediatric-onset group and the adult-onset group, the percentage of transitional B cells measured at follow up seems to be associated with immune dysregulation (AUC = 0.651 and AUC = 0.722 respectively), while CD21 low B cells do not appear to play a role (Figure 11). The sample size of the patient groups limits the interpretation of these data (pediatric-onset group *n* = 30; adult-onset group *n* = 21).

### 3.6. Associated factors to immune dysregulation in the overall cohort of CVID patients

A correlation analysis was performed between clinical features of immune dysregulation and laboratory data, particularly subsets of lymphocytes highlighted by recent studies in the literature (24–26) circulating CD8+ T cells <200 cells/µl and circulating CD21low B cells >10%. We also considered the presence of chronic EBV or CMV viremia during follow-up and the serum IgM levels at diagnosis. We found that circulating CD8+ level <200 cells/µl at follow-up correlated positively with the presence of autoimmunity (*R* = 0.313) and granulomatosis (*R* = 0.463) at follow-up, the persistence of chronic EBV viremia (*R* = 0.379) at follow-up, malignancy (*R* = 0.463) and, in particular, with lymphomas (*R* = 0.327) at follow-up, which in this series are EBV-related in 3 out of 5 subjects. Levels of circulating CD21 low B cells >10% at follow-up correlate positively with circulating CD8+ T cells <200 cells/µl (*R* = 0.327) and circulating CD4+ lymphocytes <300 cells/µl at follow-up. The presence of chronic EBV viremia is associated with the presence of autoimmunity both at diagnosis (*R* = 0.297) and at follow-up (*R* = 0.288), and it correlates with lymphoma development (*R* = 0.411). Nodular lymphoid hyperplasia showed a positive correlation with chronic EBV and chronic CMV viremia during follow-up (*R* = 0.254 and *R* = 0.550, respectively). Considering the circulating serum immunoglobulin levels measured at diagnosis, IgM was found to be inversely related to the presence of autoimmunity at diagnosis (*R* = −0.202) and follow-up (*R* = −0.218), as well as with granulomas (*R* = −0.196), GLILD (*R* = −0.287) or splenomegaly (*R* = −0.304).

## 4. Discussion

### 4.1. Age at CVID onset

Previous cross-sectional studies investigated the differences in CVID manifestations and complications according to age at onset or at diagnosis (14, 27–30). They reported that pediatric-onset CVID patients were more affected by infections and autoimmunity than adult-onset patients.

The present analysis found no significant differences in diagnosis in the prevalence of infectious disease, autoimmunity, or immune dysregulation, nor in immunoglobulin or lymphocyte profiles between patients with pediatric-onset and adult-onset CVID. Comparison at follow-up showed that patients divided by age of onset did not show significant differences in terms of initial clinical manifestations and clinical features at follow-up (except for urinary tract infections), showing that age of onset does not affect the clinical phenotype. Patients could therefore present more or less severe clinical manifestations, even immune dysregulation, regardless of the age of CVID onset. These findings are consistent with the ones recently reported by the data on the 457 patients of the USIDNET CVID cohort (15) and differ from previous reports. The USIDNET cross-sectional study on the features of patients across the age groups identified few differences between pediatric-onset and adult-onset of the disease. Some of the differences, such as bronchiectasis or granulomatous infiltration, may reflect the long-term consequences of subacute, recurrent, or chronic infections and delay in Ig replacement therapy (15). The largest cross-sectional study on 2,212 patients with CVID enrolled in ESID Registry showed also that males with CVID onset before the age of 10 years were more susceptible to pneumonia and bronchiectasis (14). Both studies did not assess longitudinal follow-up and the effects of the years of disease in the two age groups.

### 4.2. Clinical phenotypes

The present longitudinal analysis showed that during follow-up the incidence of the “only-infections” phenotype decreases and allthe patients skew to the “immune dysregulation phenotype”. The increase in immune dysregulation complications is higher inthe pediatric-onset group than in the adult-onset group. At diagnosis, 38.6% of pediatric-onset and 20.7% of adult-onset patients presented with only infections. Polyclonal lymphoid proliferation (62.1%) and autoimmunity (51.7%) were more prevalent at diagnosis in the adult-onset than in the pediatric-onset group (polyclonal lymphoid proliferation 52.3% and autoimmunity 31.8%, respectively). Enteropathy was present at diagnosis in 9.1% of pediatric-onset and 17.2% of adult-onset patients. These findings agree with those of other studies (8, 15, 16). Immune dysregulation seems to be an intrinsic feature of CVID that manifests over time. At follow-up, the sub-analysis of age at diagnosis showed that patients with pediatric-onset and adult-diagnosis manifested gastrointestinal complications more frequently than the patients with pediatric-diagnosis. A recent study comparing 9 pediatric-onset to 13 adult-onset patients with CVID found that the pediatric-onset patients with chronic diarrhea had decreased serum IgA levels and naïve CD4 T-cell and RTE cell percentages compared to the age-matched CVID patients (31).

There is increasing evidence that altered microbiome and gut barrier dysfunction contribute to systemic inflammation in patients with CVID, especially in those with lower levels of IgA and IgM antibodies binding to lipopolysaccharide and low isotype-switched memory B cells. A multi-omics approach has been used to characterize biomarkers of noninfectious complications in CVID patients (32). The data obtained are consistent with a model in which defects in humoral immunity, especially IgA, lead to barrier dysfunction, translocation of bacteria, activation of both the innate and the adaptive arms of the immune system, and tissue infiltration and/or autoimmunity.

A recent meta-analysis on neoplastic complications in patients with CVID showed an overall prevalence of malignancies equal to 8.6% (33). In our population, malignancy occurred in 12.3% of the overall cohort, divided equally between lymphomas (6.8%) and other cancers (5.5%). These data are comparable to those found in other series that report values between 1.8% and 6.7% for lymphomas and between 3% and 6.4% for other neoplasms (7, 8, 17). Lymphomas occurred similarly in both pediatric-onset and adult-onset groups.

### 4.3. Risk for immune dysregulation

In the present analysis, the risk of having a complication due to immune dysregulation in the pediatric-onset group is about double in the adult-onset patients at the same age. It may be that the longer delay in diagnosis in the pediatric-onset cohort is one of the keys to explaining their higher incidence of enteropathy due to immune dysregulation, as the prevalence of lymphoid proliferation appears to increase with time.

In the last fifteen years, many studies have focused on the description, characterization, diagnosis, and therapy of immune dysregulation in CVID, which is increasingly considered a characteristic of the disease from childhood to adulthood (7, 8, 15, 16). A meta-analysis (34) conducted on the most recent studies of the main centers that have large series of CVID patients showed the prevalence of the various conditions: autoimmune cytopenias 18.9%, gastrointestinal autoimmune diseases 11.5%, skin autoimmune diseases 5.9%, and endocrinopathies 2.5%. The prevalence of the single autoimmune complications observed in our cohort is comparable (autoimmune cytopenias 23.3%, gastrointestinal autoimmune diseases 12.3%, and skin diseases 8.2%), except for the endocrinopathies, which in our series are present in a higher percentage of patients, equal to 11.0%. We found that autoimmunity seems to increase near CVID onset in both groups by age of onset, while lymphoid proliferation and enteropathy increase over time.

A recent study conducted on 16,486 patients suffering from different inborn errors of immunity and enrolled in the ESID Registry evaluated the initial manifestations of the disease. Of the 4,244 patients affected by CVID (including the 73 patients of this cohort), the analysis described a prevalence of immune dysregulation equal to 18%, apart from neoplasms which has a total prevalence of 1% was highlighted (26). Immune dysregulation as the main manifestation at the onset of symptoms in the present overall CVID cohort is higher and reaches 42.5%. The immune dysregulation prevalence highlighted in our cohort, equal to 78%, is comparable with that of other international CVID cohorts (7, 8).

### 4.4. Biomarkers

Despite the clinical subsets of complications showing important prognostic implications, the effort to identify biomarkers of severe CVID disease for early identification is still ongoing (24). Circulating lymphocyte markers were the first extensively studied biomarkers in CVID in order to identify predisposing conditions or parameters able to predict the development of immune dysregulation. Reduced isotype-switched memory B cells and reduced T cells (CD4) can be utilized to identify those with increased complication risks. The EUROclass trial associated low switched B memory and expanded CD21 low B cell counts with splenomegaly and granulomatous disease and the expansion of transitional B cells to lymphadenopathy (25). The DEFI group found that infection-only patients had low switched memory B cell counts, while decreased naïve CD4+ T-cell counts and increased CD4 + CD95+ cells were associated with lymphoid proliferation, autoimmune cytopenias, or chronic enteropathy (26). These biomarker studies have had limited validation in pediatric cohorts (35).

We found that in the pediatric-onset group, the percentage of CD21 low B cells at diagnosis may be a reliable prognostic marker for the development of immune dysregulation during follow-up (ROC AUC = 0.796). The sample size was small, therefore the identified cut-off value (>3%) is lower than the known data in the literature (>10%) (24). In adult-onset patients, the percentage of transitional B cells measured at diagnosis showed significant accuracy (ROC AUC = 0.625) in identifying individuals at risk of developing immune dysregulation, despite the limitation of the small sample size. Similar to other previous studies (7), the association between a low number of CD8+ T lymphocytes (<200 cells/µl) and the presence of immune dysregulation and persistence of EBV viremia was also confirmed in this cohort as well as the association between persistent EBV viremia and the development of lymphoid proliferation, in particular lymphomas. EBV may promote autoimmunity not only through cross-reactivity with self-antigens, as it has been observed in SLE or multiple sclerosis, but also by expanding autoreactive age-associated B cells (ABCs) that could continuously stimulate autoreactive T cells and promote chronic inflammation in both secondary lymphoid tissue and target tissue (36).

Unlike other studies (7), no association was found between the increase in serum IgM and the development of lymphomas. In this cohort, serum IgM measured at diagnosis has a negative correlation with the development of immune dysregulation. This aspect requires further analysis and confirmatory studies.

In the latest years, genetic sequencing in CVID has been largely applied, hoping that the identification of specific genes responsible for the CVID phenotype would drive our understanding of the disease. In 10%–30% of CVID patients, pathologic monogenic defects can be identified (9–11). These defects are broadly categorized into (1) genes implicated in various stages of B cell activation, survival, or maturation to the plasma cell stage, and (2) immune-regulatory genes, with autoimmunity and inflammation (CVID) being more characteristic of the latter group. However, genetic causes of at least ∼70% of CVID patients remain unidentified to date, including those with complications (37).

Also in our cohort, targeted NGS identified a mutation in genes considered causative of CVID in 26% of patients. Among the 56 patients with complications, the majority (73.2%) did not have a causative genetic mutation. Nine of the patients with the identified gene had mutations on the TACI gene. Among these patients, seven had CVID complications. Moreover, as TACI gene variants commonly found in CVID subjects can be observed in first-degree relatives and healthy individuals with normal immunoglobulin levels, these may be more disease-modifying than disease-causing, but still, are often associated with lymphoproliferation and autoimmunity (24). The small number of patients does not allow us any further consideration.

The results of this study are consistent with other recent reports on a larger cohort of patients with CVID. They show that pediatric-onset and adult-onset do not differ in manifestation at diagnosis or follow up. Autoimmunity, cytopenia in particular, is present in close proximity to the onset of CVID, while other complications of immune dysregulation arose over time in both age-at-onset groups. Therefore, periodic monitoring with tools associated with immune dysregulation, such as CD21 low and transitional B cells, or with lymphoproliferation, such as persistent EBV viremia in the peripheral blood, appear to have a role in predicting immune dysregulation. The sample size and the data relating to the extended phenotype of lymphocyte subsets represent the main limitations of this study, therefore, our data need further investigation and validation.

In conclusion, longitudinal assessment of lymphocyte subsets combined with clinical phenotype can improve the prediction of lymphoid proliferation and allow CVID specialists to achieve earlier detection and better management of such a complex disorder. Additionally, condition-specific markers have also been suggested for lymphoma (normal or elevated IgM) and progressive interstitial lung disease (increased BAFF, normal, or elevated IgM) (24).

## Data Availability

The raw data supporting the conclusions of this article will be made available by the authors, without undue reservation.
