# Supplementary material for: Long-term follow-up in common variable immunodeficiency: the pediatric-onset and adult-onset landscape
Source: Front Pediatr. 2023 Apr 21;11:1125994. doi: 10.3389/fped.2023.1125994 (PMC10332319; doi:10.3389/fped.2023.1125994)
Supplement: Supplementary file 1 [file Table1.docx]

| **ID** | **Sex** | **Gene** | **Infection only** | **Immune Dysregulation** | **Enteropathy** | **Lymphoma** | **Autoimmunity** |
| --- | --- | --- | --- | --- | --- | --- | --- |
| #01 | M | ADA2 | **+** | **-** | **-** | **-** | **-** |
| #02 | M | ATP6AP1 | **-** | **+** | **-** | **-** | **-** |
| #03 | F | CD40 | **-** | **+** | **+** | **-** | **+** |
| #04 | M | CTLA4 | **-** | **+** | **-** | **+** | **+** |
| #05 | M | CTLA4 | **-** | **+** | **-** | **+** | **+** |
| #06 | F | CXCR4 | **-** | **+** | **-** | **-** | **+** |
| #07 | F | LRBA | **-** | **+** | **-** | **-** | **+** |
| #08 | F | NFKB1 | **-** | **+** | **+** | **-** | **+** |
| #09 | M | TACI | **-** | **+** | **+** | **-** | **+** |
| #10 | F | TACI | **-** | **+** | **-** | **+** | **+** |
| #11 | F | TACI | **-** | **+** | **-** | **-** | **-** |
| #12 | F | TACI | **-** | **+** | **+** | **-** | **+** |
| #13 | F | TACI | **+** | **-** | **-** | **-** | **-** |
| #14 | F | TACI | **-** | **+** | **-** | **-** | **-** |
| #15 | F | TACI | **+** | **-** | **-** | **-** | **-** |
| #16 | F | TACI | **-** | **+** | **+** | **-** | **-** |
| #17 | F | TACI | **-** | **+** | **-** | **-** | **+** |
| #18 | M | TNFSF12 | **+** | **-** | **-** | **-** | **-** |
| #19 | F | TTC37 | **-** | **+** | **-** | **-** | **-** |

Adjunctive table 1 _ Genetic findings and associated phenotype
