# Supplementary material for: Long-term follow-up in common variable immunodeficiency: the pediatric-onset and adult-onset landscape
Source: Front Pediatr. 2023 Apr 21;11:1125994. doi: 10.3389/fped.2023.1125994 (PMC10332319; doi:10.3389/fped.2023.1125994)
Supplement: Supplementary file 2 [file Table2.docx]

Adjunctive Table 2 _ Follow up
